# Beyond the auditory system: cognitive implications of age-related hearing loss

**DOI:** 10.3389/fnagi.2025.1736579

**Published:** 2026-01-12

**Authors:** Fabiola Paciello, Anna Pisani, Anna Rita Fetoni, Claudio Grassi

**Affiliations:** 1 Department of Neuroscience, Università Cattolica del Sacro Cuore, Rome, Italy; 2 Fondazione Policlinico Universitario A. Gemelli IRCCS, Rome, Italy; 3 Department of Neuroscience, Unit of Audiology, Università degli Studi di Napoli Federico II, Naples, Italy

**Keywords:** ageing, hearing loss, cognitive decline, mood disorders, auditory system

## Abstract

Age-related hearing loss (ARHL) is one of the most common causes of disability in older adults. It is also frequently associated with neurological and neurodegenerative disorders, including dementia, as well as with stress, anxiety, depression, and social isolation. These observations suggest that ARHL should be considered not merely as a sensory dysfunction, but rather as a complex disease involving extra-auditory domains. Namely, identifying shared pathogenic determinants between hearing loss and neurodegenerative diseases remains a significant challenge. Increasing research in this field has highlighted common molecular mechanisms underlying age-related hearing and cognitive vulnerability, as well as potential overlapping neuronal networks involved in both cognitive and auditory neurodegeneration. In this review, we first outline the clinical features, risk factors, and molecular pathways involved in ARHL. We then examine the molecular mechanisms underlying ARHL at both peripheral (cochlea) and central level (auditory cortex), and subsequently discuss the cognitive comorbidities of ARHL, with a particular focus on cognitive impairment and affective disorders. From a translational point of view, exploring the extra-auditory consequences of ARHL will be crucial, as it will enable the identification of risk factors for both auditory and cognitive vulnerability and support the development of effective therapeutic interventions.

## 1 Introduction

Age-related hearing loss (ARHL or presbycusis) is the most frequent sensory decline induced by ageing in humans (Howarth and Shone, 2006). Besides a progressive increase of auditory thresholds, starting from high frequency regions and spanning to all frequencies with advancing age, a large body of evidence demonstrates that ARHL cannot be considered merely a sensory deficit, but it can also affect cognitive functions, psychological health, frailty, or psychiatric conditions. Indeed, ARHL patients frequently experience physical and mental health dysfunctions, cognitive impairment, poor independence, social isolation, and low quality of life. Moreover, hearing loss in the elderly is considered an early landmark of dementia, including Alzheimer’s disease (AD) (Cherko et al., 2016; Cosetti and Lalwani, 2015; Taljaard et al., 2016). Clinical and experimental evidence supported the hypothesis of a strong association between hearing loss and cognitive decline (Livingston et al., 2024; Liu and Lee, 2019; Paciello et al., 2021; Paciello et al., 2023a; Paciello et al., 2023b). Indeed, hearing loss has been estimated to be one of the major modifiable risk factors for developing dementia in midlife (Livingston et al., 2024), and a substantial number of presbycusis patients show impairment in cognitive functions (Fortunato et al., 2016; Shen et al., 2018; Slade et al., 2020). Specifically, mild or moderate ARHL is often associated with deficits in working memory and executive functions (Lin et al., 2011a). Moreover, it has been shown that hearing loss is correlated to overall cognitive abilities, both factors synergistically contributing to social isolation and depression (Panza et al., 2018).

Hearing processing relies on complex cognitive functions and engages not only auditory but also “extra-auditory” brain regions to construct auditory perception. Cognitive and emotional regions of the brain play key roles in understanding speech, decoding the communicative environment, detecting potentially harmful events occurring outside the visual field, and shaping our emotional experiences of sounds. Consequently, hearing loss can have profound effects on verbal communication and significantly compromise the social, functional, and psychological well-being of individuals (Howarth and Shone, 2006; Lee, 2015; Swords et al., 2018).

In this review article, we begin by describing the clinical characteristics, risk factors, and molecular mechanisms of ARHL. Then, we focused on age-related changes in the central nervous system caused by the deterioration of auditory function, before addressing the cognitive comorbidities of ARHL, with particular attention to cognitive decline and mood disorders.

## 2 ARHL in the elderly

ARHL, also known as presbycusis, is a highly prevalent form of sensorineural hearing impairment in the elderly population. In the United States, ARHL affects approximately one-third of adults aged 61–70 years and up to 80% of those older than 85 years, accounting for nearly 60% of the total population (Lin et al., 2011b; Agrawal et al., 2008). Considering the increasing proportion of older adults, combined with environmental risk factors and unhealthy lifestyles, the prevalence of ARHL is projected to double, reaching 2.3 billion people by 2050 (Dong et al., 2025; Bainbridge and Wallhagen, 2014; Man et al., 2021). Furthermore, the World Health Organisation (WHO) predicts that by 2050, around 2.5 billion people worldwide will be affected by presbycusis (WHO, 2021).

Clinically, ARHL is characterised by mild-to-moderate bilateral hearing loss that predominantly affects high-frequency sounds, reduced speech discrimination in noisy environments, and impaired temporal and spatial auditory processing (Lee, 2015; Dubno et al., 2008; Frisina, 2009). Beyond auditory dysfunction, ARHL is strongly associated with broader health consequences in the elderly, including loneliness, social withdrawal, mood disorders, and an increased risk of cognitive decline (Lin and Albert, 2014; Panza et al., 2015; Uchida et al., 2019; Chern and Golub, 2019), thus significantly impairing the quality of life. Notably, ARHL has been identified as the leading modifiable risk factor for dementia, with the risk progressively increasing with every additional 10 dB of hearing loss (Livingston et al., 2024).

According to Schuknecht’s classification, three major clinical subtypes of ARHL can be distinguished based on both the audiometric characteristics and the nature of cochlear pathology: Agrawal et al. (2008) sensory presbycusis, defined by high-frequency hearing loss due to early degeneration of outer hair cells in the basal cochlear region; Anfuso et al. (2022) neural presbycusis, with poor speech discrimination linked to the loss of cochlear neurons and primary afferent fibres; and Akeroyd (2008) strial (or metabolic) presbycusis, with increased auditory thresholds across all frequencies and degeneration of the cochlear stria vascularis (Schuknecht and Gacek, 1993).

**Figure 1:**
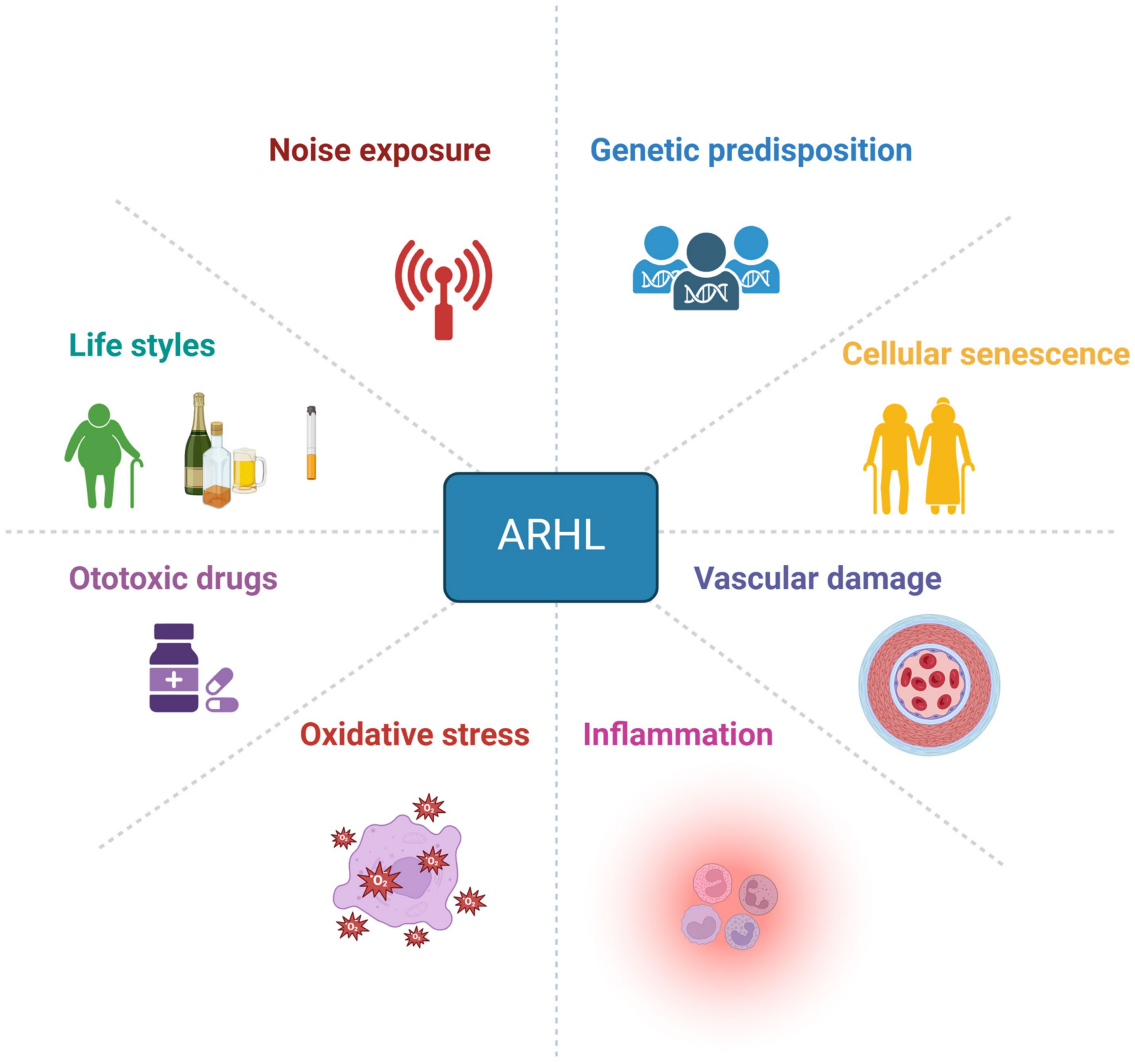
Multifactorial aetiology of ARHL. Age-related hearing loss has a complex aetiology, with multiple factors contributing to its development, including exogenous factors (noise exposure, ototoxic agents), environmental factors (unhealthy lifestyles), intrinsic factors (genetic predisposition, ageing), and concomitant mechanisms such as oxidative stress, neuroinflammation, and vascular impairment. Created with BioRender.

The mechanisms underlying the effects of ageing on different cochlear structures remain elusive (Gates and Mills, 2005), mainly due to the multifactorial aetiology of ARHL (Figure 1).

The development of ARHL is considered multidimensional, with various aspects playing a key role, including genetic predisposition and environmental risk factors (Wells et al., 2020). To date, a great contribution to our understanding of the genetic basis of ARHL has been provided thanks to the use of large-scale genome-wide association studies (GWAS) and meta-analyses (Ninoyu and Friedman, 2024). Research on animal models has contributed to identifying some candidate genes for presbycusis, including ARHL locus 1 (*Ahl1*), localised on chromosome 10, *Ahl2* (Johnson and Zheng, 2002) on chromosome 5, and *Ahl3* on chromosome 17 (Morita et al., 2007). Thus, mouse strains carrying the *Ahl* allele, such as the C57BL/6 mouse model, show early ARHL and are a well-known animal model of presbycusis (Fetoni et al., 2011; Someya et al., 2009). Amongst other genetic variants, *Gjb2* and *Gjb6*, which encode connexin 26 (Cx26) and connexin 30 (Cx30), respectively, are responsible for DFNB1, the principal cause of non-syndromic hearing loss in the Mediterranean population (Fetoni et al., 2018; Paciello et al., 2022; Xu et al., 2023).

On the other hand, environmental risk factors, such as exposure to loud noise, have been shown to strongly increase ARHL vulnerability. Indeed, exposure to high-intensity sounds, leading to noise-induced hearing loss (NIHL), during ageing has been proposed to accelerate or worsen ARHL (Gates and Mills, 2005; Kujawa and Liberman, 2006; Bielefeld et al., 2010; Fernandez et al., 2015). In the animal model of presbycusis, C57BL/6 mice, exposure to noise before the presbycusis phenotype is manifested can exacerbate cochlear senescence, leading to early ARHL, associated with increased oxidative stress and inflammatory mechanisms in the cochlea (Fetoni et al., 2022).

Taken together, owing to the multifaceted nature of the disease, our understanding of the pathogenesis of ARHL is limited, and the use of hearing aids and cochlear implants still represents the primary therapeutic strategy to improve hearing in patients with ARHL. Therefore, there is a need to further investigate the underlying pathogenesis and develop novel mechanism-based treatments.

## 3 Molecular mechanisms of cochlear ageing

Oxidative injury is presumably the principal damage associated with age-induced pathology in several organs and tissues, including the inner ear, where oxidative stress mechanisms initiate cochlear senescence (Baker and Staecker, 2012; Fujimoto and Yamasoba, 2019). Since cochlear structures have high metabolic demands, increased free radical production and oxidative stress are estimated to be major factors contributing to ARHL pathogenesis (Figure 1) (Menardo et al., 2012; Han and Someya, 2013; Benkafadar et al., 2019).

Oxidative stress implies an imbalance between the increased production of reactive oxygen species (ROS) and decreased antioxidant defence activity (Sies and Jones, 2020). This can result in cell damage due to the oxidation of cellular components, such as membrane lipids, proteins, and DNA (Serrano and Klann, 2004). In previous studies, we documented how increased ROS production in the cochlea, induced by several exogenous factors, including noise exposure or ototoxic drugs, can lead to lipid peroxidation (Fetoni et al., 2013; Fetoni et al., 2016). In particular, lipid peroxidation in the outer hair cells (OHCs) affects the plasma membrane fluidity, altering their motility and amplifying properties, thus contributing to functional damage after noise exposure (Maulucci et al., 2014).

Mitochondria generate ROS through the mitochondrial respiratory chain, which produces oxidants as part of aerobic respiration (Sies and Jones, 2020). Thus, mitochondria are the major source of ROS during ageing (Xu et al., 2025). Overproduction of free radicals leads to mitochondrial dysfunction and an associated decrease in energy production in cochlear cells (Balaban et al., 2005; Henderson et al., 2006; Lin and Beal, 2006). ROS initiates the mitochondria-mediated apoptotic cascade by cytochrome-c release, the activation of the initiator caspase-9, and its effector caspase-3, leading to cell death (Chen et al., 2003). Moreover, oxidative stress can damage mitochondrial components, such as respiratory chain proteins, mitochondrial membranes, mitochondrial DNA (mtDNA), and nuclear DNA, which affect mitochondrial and cellular function (Guo et al., 2013).

The central role of ROS in cochlear senescence has also been supported by evidence showing the role of several proteins involved in redox signalling in determining susceptibility to develop ARHL. Amongst these, the adaptor protein p66shc has been suggested to function as a redox enzyme. Under physiological conditions, it is located in the cytosol of the cells; however, when ROS production increases, it translocates to the mitochondria, where it serves as an important source of ROS by oxidising cytochrome c and generating H_2_O_2_ (Giorgio et al., 2005; Pinton et al., 2007). Cells lacking the p66shc gene show a reduced number of free radicals and increased tolerance to oxidative stress (Migliaccio et al., 1999). Moreover, knockout (KO) mice lacking the p66shc gene are resistant to several age-related diseases (Berniakovich et al., 2008; Napoli et al., 2003; Ranieri et al., 2010), including cochlear damage (Fetoni et al., 2016). Indeed, p66shc KO mice are resistant to noise-induced hearing loss, and they show a delayed onset of ARHL with advancing age, with decreased levels of oxidative stress and vascular damage in cochlear structures (Fetoni et al., 2016).

Someya et al. (2010) highlighted the role of sirtuins, specifically SIRT3, in cochlear ageing, showing that SIRT3 KO mice have accelerated ARHL compared to wild-type animals (Someya et al., 2010). Furthermore, the protective role of sirtuins in cochlear ageing was also supported by evidence showing that silencing of SIRT1 led to cell death due to autophagy inhibition, whereas activating SIRT1 and autophagy reversed cell death by modulating the deacetylation of the ATG9A autophagy protein (Pang et al., 2019). In addition to promoting autophagy, sirtuin signalling can also boost the antioxidant defence system, counteracting cochlear redox imbalance in several models of cochlear injury (Pisani et al., 2025; Singh et al., 2018; Someya et al., 2010; Zheng et al., 2025).

Other mechanisms closely associated with ageing and ARHL include autophagy (Fujimoto et al., 2017). Indeed, accumulating evidence suggests a significant relationship between autophagy and senescence, as the stimulation of autophagy has been linked to promoting longevity (Leidal et al., 2018). Autophagy is a pivotal intracellular degradation pathway (Wu et al., 2020) that involves the encapsulation and transport of compromised organelles and abnormally large molecules to lysosomes for degradation. MicroRNAs (miRNAs) are emerging as significant contributors to autophagy (Huang et al., 2021; Li et al., 2022; Qian et al., 2021). In particular, studies have highlighted the role of different miRNAs, including miR-34a, which targets ATG9A, and miR-489, which acts as a negative regulator of NDP52, in ARHL pathogenesis (Pang et al., 2017; Li et al., 2022). Additionally, miR-34a a/SIRT1 exerts protective effects by counteracting cochlear oxidative stress through stimulating mitochondrial autophagy (Xiong et al., 2019). However, the exact role of autophagy in ARHL pathogenesis remains elusive and requires further investigation.

Oxidative stress is also strongly related to vascular dysfunctions. ROS can indeed activate the vascular endothelial growth factor (VEGF), playing a crucial role in angiogenesis and vascular repair. Cochlear VEGF expression has been reported to be affected in animal models of hearing loss induced by noise, ototoxic drugs, or ageing (Fetoni et al., 2009; London and Gurgel, 2014; Fetoni et al., 2022; Anfuso et al., 2022). Specifically, experimental evidence showed decreased expression in cochlear structures of animals with presbycusis, indicating that vascular abnormalities may contribute to ARHL aetiology (Picciotti et al., 2004). Moreover, the exposure to loud noise, considered an environmental risk factor for ARHL, causes increased vascular permeability, affecting cochlear blood flow (Shi, 2009). Additional studies suggest a VEGF upregulation in the cochlea of animals with NIHL (Picciotti et al., 2006), suggesting that an enhanced VEGF level can be considered an endogenous mechanism trying to face cochlear injury.

Taken together, experimental evidence demonstrates multiple co-occurring mechanisms of action underlying cochlear injury in ARHL. However, the causal relationships amongst these factors remain unclear, and further studies are needed to elucidate their specific contributions.

## 4 Central auditory dysfunctions in ARHL

The process of auditory perception in response to sound involves the transformation of a mechanical stimulus (vibration of inner ear structures induced by sound waves) into electrical signals (action potentials) within the cochlear sensorineural epithelium. The stereocilia, located at the top of hair cells, are essential for auditory mechanical transduction. When sound waves enter the inner ear, they cause the vibration of the basilar membrane, running from the basal to apical region of the cochlea and containing the three rows of OHCs and one row of inner hair cells (IHCs). This vibration results in the movement of the tectorial membrane and the deflection of the stereocilia, leading to the opening of K^+^ channels, causing cell depolarisation (Musiek and Baran, 2018). This triggers neurotransmitter release, generating the electrical signals that, through spiral ganglion neurons (SGNs) fibres, spread to the nuclei of the auditory brainstem to reach the auditory cortex through the medial geniculate afferent fibres (Musiek and Baran, 2018).

However, behind sound processing, the auditory perception also involves a complex mechanism to decode and comprehend the auditory message, so that our ability to detect, localise, and identify sounds exceeds the simple transduction of sensory input (Paciello et al., 2023b). Auditory brain structures can construct hearing perception, using a combination of information learned during development (Litovsky, 2015). This process also involves other extra-auditory brain regions, leading to the construction and analysis of the auditory scene (sound patterns by different sources are combined in a coherent perception), the perception and coding of pitch, understanding language, appreciating music, focusing on the speech in a noisy environment, and so on (Humes et al., 2013a).

Ageing processes can affect all these abilities, leading to both functional and synaptic reorganisation within the auditory system, both in the periphery and in the CNS (Willott et al., 1993; Lin and Albert, 2014).

**Figure 2:**
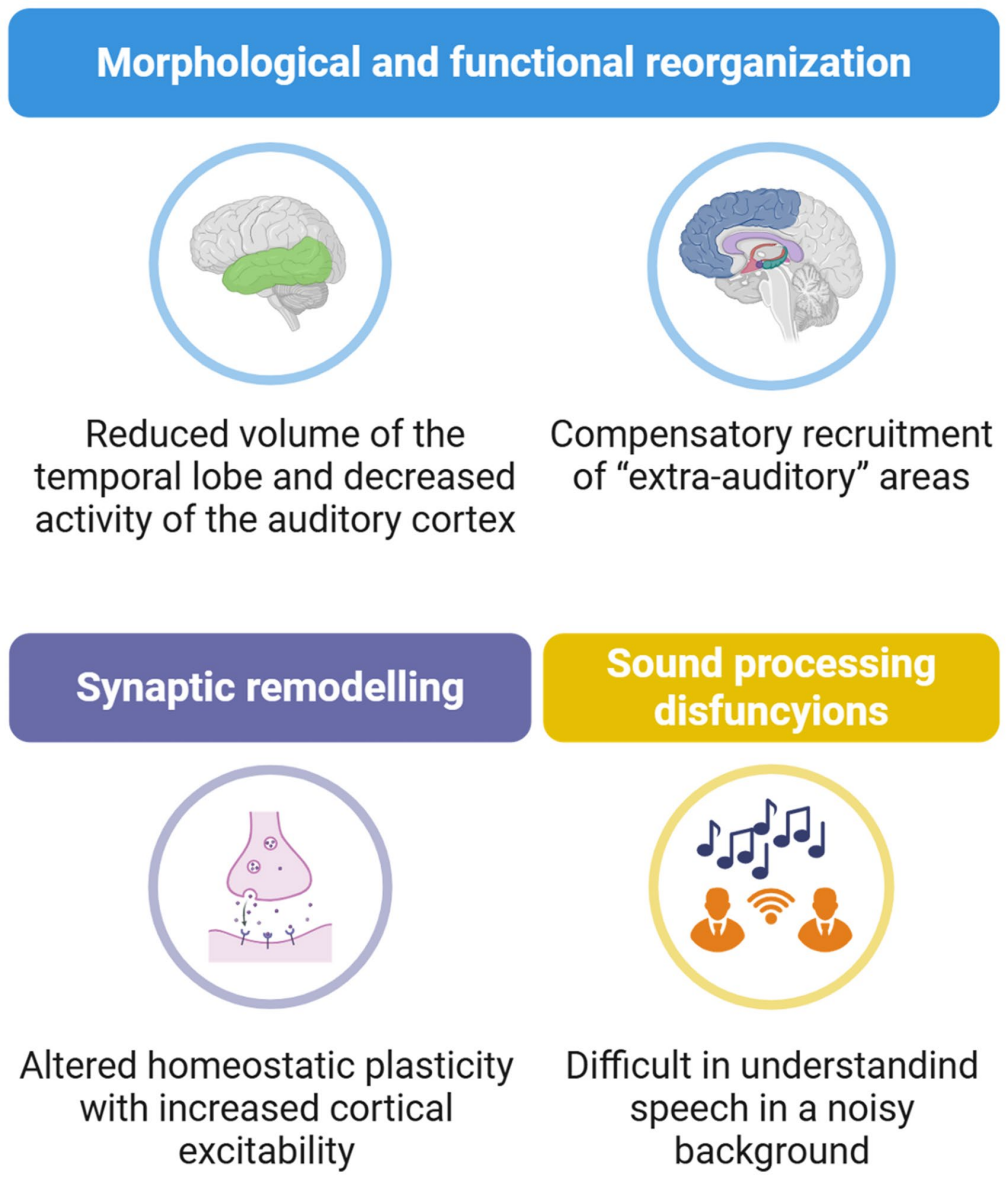
Central auditory dysfunctions in ARHL. Hearing impairment during ageing is associated with a profound reorganisation of brain networks, brain plasticity alterations, and functional outcome. ARHL is characterised by reduced activity in the auditory cortex and atrophy of the temporal lobe, along with a compensatory recruitment of extra-auditory regions, including the prefrontal cortex and limbic system. At the cellular level, an imbalance between excitatory and inhibitory synapses has been observed in auditory structures. Altogether, these cortical modifications lead to altered auditory processing and difficulties in speech comprehension, particularly in noisy environments. Created with BioRender.

Indeed, ARHL has been associated with a general decreased brain volume of the temporal lobe, with increasing difficulties in presbycusis patients to understand speech in a noisy environment (Figure 2) (Fitzgibbons and Gordon-Salant, 2010; Humes and Dubno, 2021). Interestingly, patients with hearing impairment show lower activity in auditory structures, associated with an increased compensatory recruitment of “extra-auditory” areas, not usually involved in speech processing, including the prefrontal cortex and the cingulo-opercular circuit (Figure 2), supporting the idea of an extended brain network, behind auditory pathway, to sustain language processing in presbycusis patients (Peelle and Wingfield, 2016). Specifically, auditory deprivation results in decreased intra-modal activation of the auditory cortex in presbycusis patients, likely impairing the ability to discriminate language in a noisy background. In this context, visual perception becomes crucial, as patients rely on visual cues to disambiguate the degraded speech signal. Thus, at the cortical level, a cross-modal recruitment by vision appears to occur in ARHL (Campbell and Sharma, 2013; Campbell and Sharma, 2014).

Amongst extra-auditory regions, the cingulo-opercular cortex has drawn particular attention. This brain area, which also contributes to speech comprehension in normal hearing subjects, shows signs of atrophy in ARHL patients, especially in those with concomitant episodic memory deficits (Belkhiria et al., 2019). These findings suggest a potential involvement of the Papez circuit in ARHL, highlighting the interplay between auditory dysfunction and brain regions implicated in cognitive functions.

Additionally, several evidence also support a reorganisation of homeostatic plasticity, leading to maladaptive activity-dependent changes in neuronal activity (Figure 2). Indeed, a decreased level of GAD65 and GAD67, two enzymes involved in GABA synthesis, was found in the auditory cortex of two different strains of aged Wistar rats (Ling et al., 2005; Burianova et al., 2009), along with a decreased number of paravalbumin-positive neurons (Ouda et al., 2008). Consistently, patients with ARHL show increased wave amplitude of cortical auditory evoked potentials, suggesting increased cortical response to sound, probably related to loss of inhibition (Schmidt et al., 2010; Herrmann and Butler, 2021). Another crucial aspect in this context is the redistribution of membrane-bound ion channels, which modulate the ion conductance and, thus, the excitability of neurons (Jung et al., 2005). Calcium-dependent activity was significantly reduced in higher auditory structures in ageing mice (Gröschel et al., 2014). Interestingly, similar findings were found in the hippocampus, a structure playing an important role in neuroplasticity and memory formation, and probably further involved in tinnitus and related hearing disorders (De Ridder et al., 2006; Kraus and Canlon, 2012). Of note, older adults with preserved robust cognitive performance show lower cortical excitability compared to less cognitively robust older adults, suggesting that their lower excitability is associated with higher cognitive function (Cespón et al., 2022).

In line with these considerations, boosting neuronal activity by using noninvasive neuromodulator techniques, such as anodal direct current stimulation in the auditory cortex, can delay central ARHL by minimising the loss of inhibition and by preventing increases in cortical excitability in Wistar rats (Fernández del Campo et al., 2025).

## 5 Hearing loss and cognitive decline

### 5.1 Current hypotheses

Consistent evidence from epidemiological studies strongly suggests an association between hearing loss and cognitive decline (Gallacher et al., 2012; Thomson et al., 2017; Livingston et al., 2020; Loughrey et al., 2018; Liu and Lee, 2019), with this link becoming more pronounced with ageing (Lindenberger and Baltes, 1994; Fortunato et al., 2016). Specifically, hearing loss has been linked to incidence and acceleration of cognitive deficits (Bernabei et al., 2014; Amieva et al., 2015; Fortunato et al., 2016; Deal et al., 2017), as well as to increased risk for the onset of neurodegenerative disorders, including Alzheimer’s disease (AD) (Gates et al., 2002; Panza et al., 2015; Taljaard et al., 2016; Shen et al., 2018). Accordingly, recent evidence demonstrates that patients affected by hearing loss and treated with hearing aids or cochlear implants show an improvement of cognitive functions, including executive function, visuospatial abilities and verbal memory, with variability in cognitive outcomes depending on the type and severity of hearing loss (Gates et al., 2002; Panza et al., 2015; Taljaard et al., 2016; Shen et al., 2018). Similarly, it has been shown that patients wearing hearing aids were less likely to develop mild cognitive impairment (MCI), a transitional stage between healthy ageing and dementia, than hearing-impaired individuals who did not use hearing devices (Bucholc et al., 2022). Thus, given that hearing loss represents the most modifiable risk factor for developing dementia, research in this field has grown substantially in recent years.

**Figure 3:**
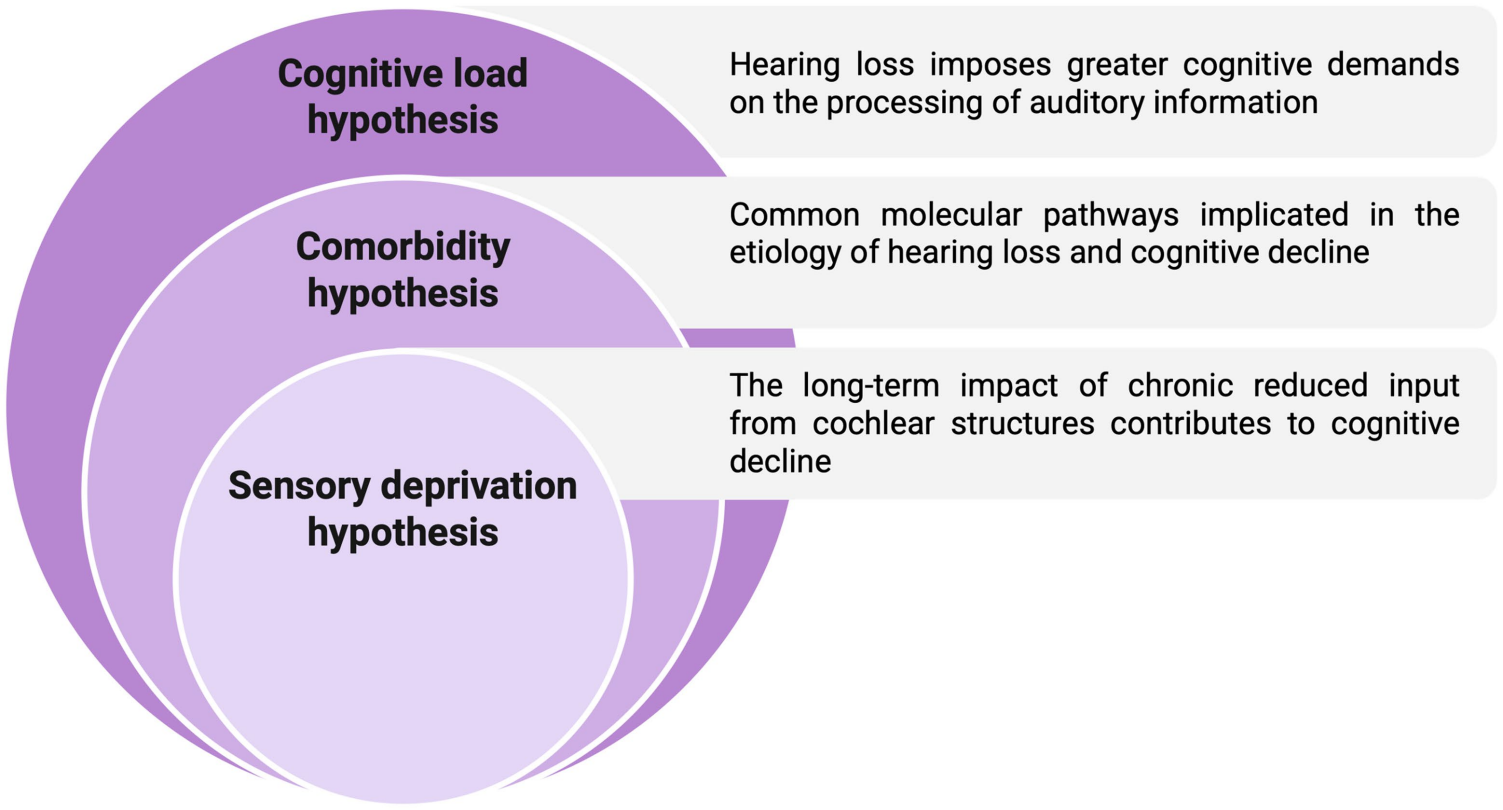
Possible mechanisms relating ARHL and cognitive decline. Three main mechanisms have been proposed to explain the link between hearing loss and cognitive decline: the “cognitive load hypothesis,” the “comorbidity hypothesis,” and the “sensory deprivation hypothesis”.

Currently, three hypotheses have been proposed to explain the link between hearing sensitivity and cognitive functions: the “cognitive load hypothesis,” the “comorbidity hypothesis,” and the “sensory deprivation hypothesis” (Figure 3). Specifically, according to the cognitive load hypothesis, when people with hearing loss have difficulties in understanding speech or other sounds, their cognitive system must allocate extra resources to processing these auditory signals. This additional demand can impact the overall cognitive load, leading to cognitive decline over time (Humes et al., 2013b). Comorbidity hypotheses suggest the existence of a common cause, based on shared molecular mechanisms underlying hearing loss and cognitive decline. In this scenario, pathological markers, including decreased brain volume, oxidative stress, neuroinflammation, vascular dysfunction or increased accumulation of pathological markers, including *β*-amyloid and tau phosphorylation, play a key role (Zhao et al., 2024; Griffiths et al., 2020). Finally, based on the sensory deprivation hypothesis, the long-term impact of chronic hearing deficit and decreased input from cochlear structures leads to cognitive decline. This is due to compensatory cortical neuronal processes impacting cognitive processes (Zhao et al., 2024).

### 5.2 ARHL and dementia

In the context of ARHL, two main factors must be considered: Agrawal et al. (2008) cognitive reserve progressively declines with ageing, and Anfuso et al. (2022) hearing loss increases the demand for cognitive resources, language skills, and other higher-order abilities required for listening and comprehension, thereby raising the overall cognitive load. Indeed, ARHL is linked to an increased risk of the onset of dementia (Albers et al., 2015). Amongst cognitive abilities required for language processing, working memory plays a crucial role, and it has been linked to ARHL (Janse and Jesse, 2014; Nagaraj and Atcherson, 2017). Indeed, working memory allows the temporary storage and manipulation of information necessary for complex cognitive processes, including language comprehension, learning, and reasoning (Baddeley, 2003). Thus, working memory represents a cognitive resource supporting language processing, especially in situations where speech comprehension is challenging, such as in noisy environments (Dryden et al., 2017; Moore et al., 2014; Marsja et al., 2022). Namely, a strong reduction of working memory occurring during ageing is closely associated with auditory processing abilities (Akeroyd, 2008; Mukari et al., 2014; Humes et al., 2022), particularly in tasks requiring speech recognition in noisy environments (Pichora-Fuller et al., 1995; Javanbakht et al., 2021; Arjmandi and Behroozmand, 2024). Moreover, ARHL patients show impaired working memory compared to those with normal hearing (Madashetty et al., 2024; Chang and Chen, 2025; Shin et al., 2025). These findings suggest that increased memory demands negatively affect the speech recognition abilities of older adults (Gordon-Salant and Fitzgibbons, 1997).

Additionally, data from animal models suggest that hearing loss can affect brain structures involved in cognitive functions, such as the hippocampus, by altering neurotransmitter levels (Cui et al., 2009; Chengzhi et al., 2011; Beckmann et al., 2020) and by decreasing neurogenesis (Tao et al., 2015; Liu et al., 2016; Kurioka et al., 2021). Moreover, we previously demonstrated that hearing loss can accelerate cognitive decline in an animal model of AD (the 3 × Tg-AD mice), causing early recognition memory impairment together with morphological and functional alterations in the hippocampus. At the molecular level, increased oxidative stress, neuroinflammation, and accumulation of AD markers (such as tau phosphorylation) were found in the hippocampus of 3 × Tg-AD mice with hearing loss and exhibiting early cognitive decline (Paciello et al., 2021). Similarly, worsening hearing impairment in a model of early ARHL (the C57BL/6 mouse strain) caused deficits in working memory performance, associated with increased oxidative stress and inflammation in the hippocampus (Paciello et al., 2023a). Other studies suggest that auditory experience can impact hippocampal functions: Martorell et al. (2019) demonstrated that trains of tones repeating at 40 Hz (gamma frequency) drive gamma activity in the auditory cortex and the hippocampus, leading to improved behaviour and decreased amyloid deposition in the hippocampus of the 5 × FAD AD mouse model (Martorell et al., 2019). On the other hand, auditory nerve hyperexcitability, leading to SGN degeneration and ARHL, was also found in 5 × FAD mice (Su et al., 2025), suggesting a bidirectional link between auditory deficit and cognitive neurodegeneration.

About the molecular mechanisms underlying the association amongst ageing, hearing loss, and dementia, recent data support the role of shared damaging mechanisms (Griffiths et al., 2020), such as oxidative stress and inflammation or vascular damage (Paciello et al., 2023b).

Despite considerable interest in this field, further studies are needed to clarify the molecular and cellular mechanisms linking hearing loss to cognitive decline and to determine how reduced auditory function impacts cognitive abilities.

## 6 ARHL and mood disorders

Globally, more than 320 million people suffer from depression, and more than 260 million people experience anxiety disorders, representing the primary causes of disability worldwide (WHO, 2021). ARHL and mood disorders, including depression, anxiety, and stress, share the same risk factors in the elderly (Monzani et al., 2008). Moreover, a high grade of comorbidity exists, with presbycusis patients exhibiting various health outcomes similar to those associated with depression, anxiety, and stress, including reduced quality of life, low levels of physical activity, frailty, social isolation, and poor general health (Khalsa, 2015; Livingston et al., 2017). Consequently, ARHL, depression, and anxiety are considered amongst the leading causes of disability worldwide and may mutually promote each other’s occurrence (Jayakody et al., 2018).

The strong relationship between auditory and limbic systems is supported by the notion that acoustic stimuli processed along the auditory pathway undergo an emotional labelling, so that acoustic signals can be classified and perceived with positive or negative emotive valence. This is possible due to the neuronal connectivity between auditory and limbic circuits in the amygdala, hippocampus, and prefrontal cortex regions (Kraus and Canlon, 2012). Thus, the auditory/limbic networks are involved in detecting adversity, danger, and in the regulation of the hypothalamic–pituitary–adrenal (HPA) axis, the major neuroendocrine system controlling reactions to stress (Dunlavey, 2018).

Clinical evidence shows that poorer ability to understand language and communicate, especially in complex social situations, represents a source of stress and mental fatigue for ARHL patients. This can lead to social isolation, predisposing individuals to develop mood disorders, such as depression, anxiety, and stress symptoms (Mener et al., 2013; Peelle and Wingfield, 2016).

Several studies have reported the association between depression and hearing loss, with a causal role of hearing impairment in determining depression (Kalayam et al., 1991) and anxiety (Jayakody et al., 2017). It has been shown that the severity of hearing impairment, regardless of whether speech or high-frequency losses were assessed, is linked to the severity of mental health symptoms (Jayakody et al., 2018). A relationship between the onset of depression and stressful life events has also been reported. Exposure to both physical and psychosocial stressors, including acoustic trauma, ototoxic drug administration, and sleeping problems, is related to ARHL. Previous studies implied that pharmacological or acoustic trauma-induced stress affects central auditory processing through sensorineural cochlear responses (Singer et al., 2013; Singer et al., 2018).

Moreover, there are overlapping neurological, anatomical, and physiological processes involved in mood disorders and ARHL. Specifically, changes in grey matter volume of several brain regions were associated with mental health symptoms in ARHL patients. Indeed, apathy has been linked to reduced volumes of the insula and amygdala (Belkhiria et al., 2020), anxiety was associated with reduced volume of the superior and medial frontal gyrus (Husain et al., 2011; Boyen et al., 2013), and a significant association between high-frequency hearing loss and anxiety scores was found in ARHL patients, showing also decreased volume of the hippocampal/para-hippocampal regions and of the middle cingulate cortex (Ma et al., 2022). This evidence supports the idea that ARHL and mood disorders probably share common neuronal pathways and are strongly related.

Controversial evidence has been reported regarding the role of corticosteroid signalling in the auditory system, with some studies suggesting a protective role on the auditory system (Meltser and Canlon, 2011; Lee et al., 2019) and others suggesting a detrimental role, demonstrating an association between stress and auditory hallucinations, sudden sensorineural hearing loss, Menière’s disease, hyperacusis, and misophonia (Mazurek et al., 2012; Guetta et al., 2024; Horner and Cazals, 2005).

Corticosteroid receptors, including mineralocorticoid (MR) and glucocorticoid receptors (GR), are expressed in the inner ear and in the auditory structures of the CNS (Kil and Kalinec, 2013; Terakado et al., 2011). Experimental studies showed that chronic stress and increased glucocorticoid levels in response to noise exposure can trigger early signs of cognitive impairments, anxiety-like behaviours, and AD-like neuropathological changes throughout the lifespan (Jafari et al., 2019a; Jafari et al., 2019b). Clinical observations have suggested a relationship between emotional stress and auditory disturbances, such as tinnitus, characterised by the perception of phantom sounds without external stimuli (Henton and Tzounopoulos, 2021), and hearing loss or presbycusis at an early age (Pérez-Valenzuela et al., 2019). Proposed theories indicate that the HPA axis may play a role in influencing neuroplasticity and glutamatergic neurotransmission (Guetta et al., 2024). Short-term corticosteroid exposure generally increases AMPA glutamate receptor (AMPAR) localisation and mobility in neuronal cultures, but results from *in vivo* models have shown significant variability due to differences in exposure conditions, species, and stress responses (Edlund et al., 2025).

On the other hand, it is thought that glucocorticoids have protective effects against hearing loss via GR, because of their anti-inflammatory and immunosuppressive action, whilst the aldosterone-selective MR are involved in the maintenance of ion homeostasis required for hearing sensitivity (Meltser and Canlon, 2011). Indeed, it has been shown that the functional role of GR and MR is in regulating the synapses between IHCs and primary afferent fibres (Marchetta et al., 2022). This suggests that either limbic MR or GR function contributes to the precision of auditory processing, thereby possibly influencing speech comprehension and cognitive auditory processing (Marchetta et al., 2022). Further, the positive action of corticosteroids on the auditory system is supported by the therapeutic use of synthetic corticosteroids (prednisone, dexamethasone) to treat inner ear dysfunctions, such as sudden sensorineural hearing loss or tinnitus (Hobson et al., 2016; Leung et al., 2016; Barreto et al., 2012; Dodson and Sismanis, 2004). However, whilst clinical evidence has shown the effectiveness of glucocorticoids for the treatment of various inner ear diseases, their mechanisms of action and the optimal timing of treatment are not well understood (Lee et al., 2019; Meltser and Canlon, 2011).

Collectively, this evidence confirms a strong relationship between mental health and hearing loss in the elderly, with bidirectional effects and shared neurological features.

## 7 Concluding remarks

**Figure 4:**
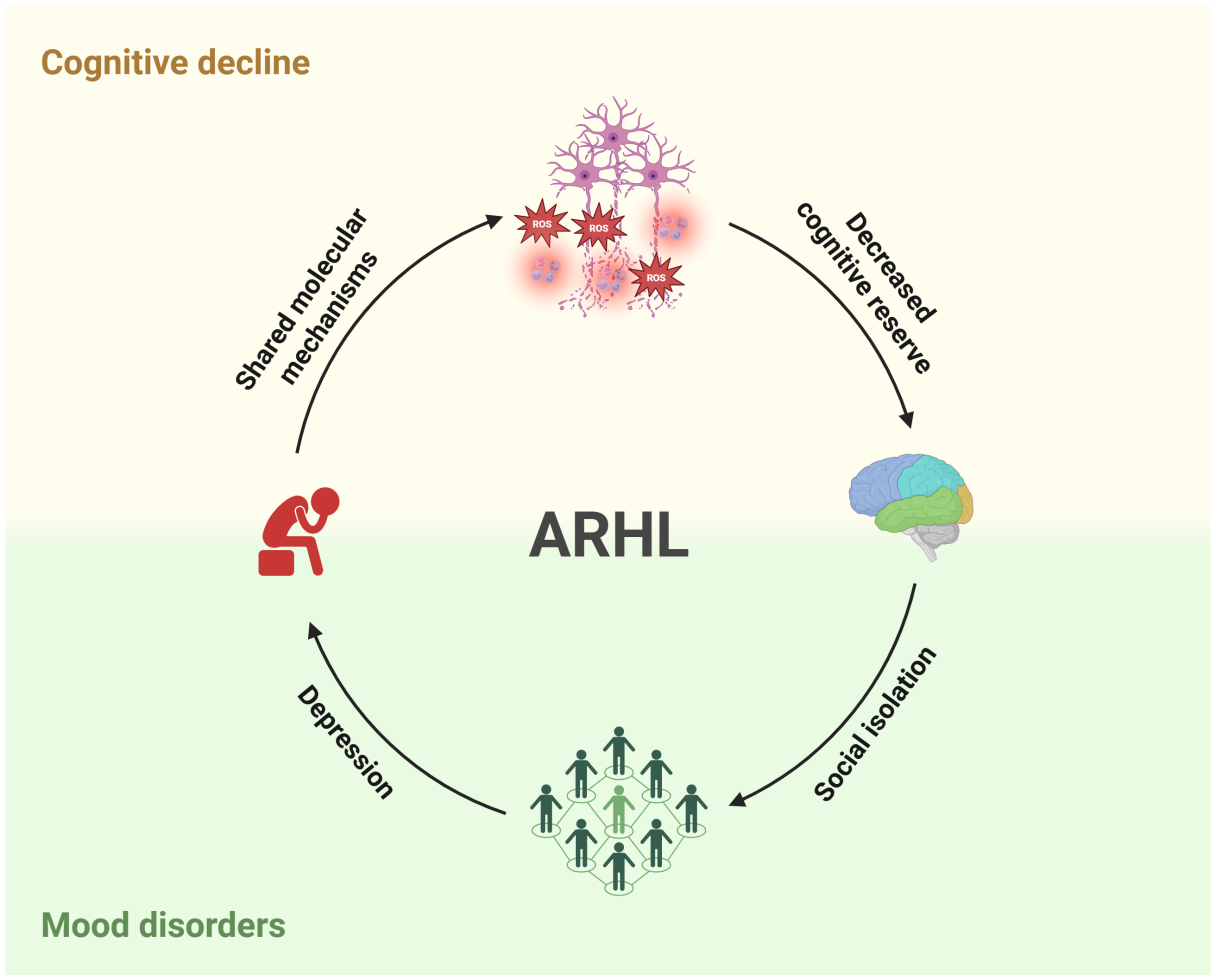
Complex relationship between ARHL, cognitive decline, and mood disorders. Schematic illustration showing how cognitive dysfunctions and mood disorders are strongly linked to ARHL shared the same molecular mechanisms and psychological vulnerability, exacerbating the quality of life of patients affected by hearing loss. Created with BioRender.

ARHL is a multifactorial disease with different etiopathological sources, including genetic susceptibility, lifestyles and exposure to risk factors. Both clinical and experimental evidence confirmed a strong association between hearing impairment and neurological diseases, including cognitive decline and mood disorders (Figure 4). Recent evidence demonstrated shared molecular mechanisms affecting both hearing functions and cognitive reserve, including oxidative stress, neuroinflammation, and vascular damage. Moreover, hearing and cognitive functions seem to share the same brain networks, spanning from the auditory cortex to the hippocampus and limbic system.

Notwithstanding the growing interest in this field, due to the fact that acting on hearing loss can counteract brain neurodegeneration, the complex mechanisms underlying the relationship between auditory and cognitive functions are still elusive and need further investigation. From a translational point of view, this is an intriguing research field, allowing us to target risk factors of auditory and cognitive vulnerability and develop effective therapeutic interventions.
